# Effect of Chemical Stabilizers on the Thermostability and Infectivity of a Representative Panel of Freeze Dried Viruses

**DOI:** 10.1371/journal.pone.0118963

**Published:** 2015-04-29

**Authors:** Boris Pastorino, Cecile Baronti, Ernest A. Gould, Remi N. Charrel, Xavier de Lamballerie

**Affiliations:** Aix Marseille Université, IRD French Institute of Research for Development, EHESP French School of Public Health, EPV UMR_D 190 "Emergence des Pathologies Virales", & IHU Institute hospitalo-universitaire Méditerranée Infection, APHM Public Hospitals of Marseille 13385, Marseille, France; University of Edinburgh, UNITED KINGDOM

## Abstract

As a partner of the European Virus Archive (EVA) FP7 project, our laboratory maintains a large collection of freeze-dried viruses. The distribution of these viruses to academic researchers, public health organizations and industry is one major aim of the EVA consortium. It is known that lyophilization requires appropriate stabilizers to prevent inactivation of the virus. However, few studies have investigated the influence of different stabilizers and lyophilization protocols on the thermostability of different viruses. In order to identify optimal lyophilization conditions that will deliver maximum retention of viral infectivity titre, different stabilizer formulations containing trehalose, sorbitol, sucrose or foetal bovine serum were evaluated for their efficacy in stabilizing a representative panel of freeze dried viruses at different storage temperatures (-20°C, +4°C and +20°C) for one week, the two latter mimicking suboptimal shipping conditions. The Tissue Culture Infectious Dose 50% (TCID_50_) assay was used to compare the titres of infectious virus. The results obtained using four relevant and model viruses (enveloped/non enveloped RNA/DNA viruses) still serve to improve the freeze drying conditions needed for the development and the distribution of a large virus collection.

## Introduction

EVA (European virus Archive) is a non-profit organization, funded by the European Commission through the FP7 CAPACITIES Project GA n° 228292. EVA mobilizes a European network of scientific laboratories with expertise in virological procedures to collect, characterize and distribute viruses and virus-derived products such as antibodies, antigens, diagnostic and research reagents and assay [1]. As the coordinating partner of EVA, we have propagated a large number of virus strains, for worldwide distribution. Although ultra-low temperature storage at -80°C appears easy and cost effective, the potential problems involved in long-term preservation (i.e. the possibility of power/equipment failure) in the source laboratory, together with shipping to the customer (such as costs, packaging and cold chain preservation) need to be developed to the safest, highest, most robust and cost effective standards. Cryopreservation has been robustly demonstrated as a means of preserving a wide range of microorganisms and lyophilization in particular is known to preserve viruses for sixty or more years [2] but few studies have evaluated and compared different lyophilization protocols for the efficient preservation of various categories of viruses (enveloped/non-enveloped, RNA/DNA genome). Preparation of the infectious virus samples before and during lyophilization is critical for the quality of the end-product. For example, a controlled rate of freezing of each virus sample and the use of small volumes of virus in each aliquot can significantly improve survival of virus infectivity. The use of appropriate stabilizers can also greatly reduce loss of infectivity arising from damage to virus structural proteins and nucleic acids resulting from temperature changes and stresses imposed by crystallization and solubilisation, during the lyophilization and recovery protocols [3–4].

Lyophilization of cells in water or a simple salt solution typically results in poor survival. Consequently, using the same assumptions, a variety of protective media has been developed for lyophilization of viruses, including augmented growth media or sugar, and peptide solutions [5]. Most common stabilizer formulations include carbohydrates (sucrose, lactose, maltose, and trehalose) or sugar alcohols (such as sorbitol and mannitol) [6–7]. Carbohydrates are widely used as cryoprotectants based on pragmatic grounds rather than evidence-based data but disaccharides are reported to be more effective than monosaccharides when used in conventional freeze-drying protocols since they display higher collapse [8]. Alternatively, reducing sugars may induce damaging Maillard reactions, thereby compromising stability, and for this reason non-reducing disaccharides, such as sucrose or trehalose, are preferred to reducing disaccharides such as lactose.

The freeze drying protocol can also determine the properties of a specific formulation by influencing the crystallization of additives and subsequently acting on the collapse temperature. It is known that significant crystallization of the bulking agent will decrease drying time but can also reduce stabilizing effects of the amorphous stabilizer especially with proteins. In fact, different freezing or drying rates can produce vastly different freeze-dried products, both in appearance and viability of the micro-organism and an optimized lyophilization protocol will provide excellent drying efficiency while maintaining the virus integrity [9]. Although lyophilization having been used for decades, the conservation of biological products is affected by so many factors that it is often necessary empirically to adjust the experimental conditions for each particular case. However, few experimentally-based data are available in the scientific literature to be applied easily for the development and the distribution of a large collection of viruses. In fact, specific protocols are required to reduce the damage induced by preservation techniques or storage conditions.

This study focuses on optimizing both the protocol and the formulation for the lyophilization of enveloped and non-enveloped viruses possessing RNA or DNA genomes. One aim was to select the standardized method allowing optimal recovery of viral infectivity after one week at +4°C or +20°C, conditions mimicking transportation without cold chain preservation. The performance of the protocols is evaluated by measurement of the infectivity of the resulting freeze-dried material. Using a representative panel of viral specimens, several chemical stabilizers were assayed to evaluate the thermostability of the freeze-dried preparation. Commonly known cryo/lyoprotectants, including sucrose, trehalose, sorbitol or foetal bovine serum in two freeze-drying programmes, were compared for their stabilizing property on four representative viruses. After different temperature storage conditions representing those that may be encountered during viral transport and distribution, the recovery of viral infectivity titre was analyzed to identify the optimal conditions for the lyophilization protocol.

## Materials and Methods

### 1. Cells and viruses

The four virus strains used in this study were isolated from human clinical samples collected for diagnosis in the Virology laboratory of the University Hospital of Marseille (Table 1). These strains are available from the EVA collection (European-virus-archive.com).

**Table 1:** Representative RNA viruses used in this study.

	Genome	Family	Genus	Virus	Strain	Cell line
**Enveloped viruses**	ssRNA	Togaviridae	Alphavirus	Chikungunya virus	LR2006_OPY1	Vero
	dsDNA	Herpesviridae	Simplexvirus	Herpesvirus type 1	MRS2010 7351047	MRC-5
**Non Enveloped viruses**	ssRNA	Picornaviridae	Enterovirus	Human Echovirus 13	MRS 142604/2000	MRC-5
	dsDNA	Adenoviridae	Mastadenovirus	Human Adenovirus type C	MRS2012 23910121	Hep-2

Human herpesvirus 1 (strain MRS2010 7351047) and Human enterovirus B type Echovirus 13 (strain 142604/2000) were propagated on primary human fibroblast MRC-5 cells (ATCC number CCL-171) grown in Basal Medium Eagle (BME) supplemented with 10% foetal calf serum (FCS), 2 mM L-glutamine, 100U/ml Penicillin, 100μg/ml Streptomycin sulfate, under 5% CO2. Chikungunya virus (strain LR2006_OPY1) was propagated on Vero cells (ATCC number CCL-81) grown in minimal essential medium (MEM) supplemented with 5% FCS, 2 mM L-glutamine,100U/ml Penicillin, 100 μg/ml Streptomycin sulfate, under 5% CO2. Human Adenovirus type C (strain MRS2012 23910121) was propagated on a Hep-2 cell line (ATCC number CCL-23) grown in minimal essential medium (MEM) supplemented with 5% FCS, 2 mM L-glutamine,100U/ml Penicillin, 100 μg/ml Streptomycin sulfate, 1% non essential amino-acids, under 5% CO2.

### 2. Stabilizers

Four different stabilizer solutions were tested in this study. For each, two concentrations were prepared depending on the data found in the literature. Sorbitol, trehalose and sucrose were obtained from Sigma (France). foetal bovine serum (FBS) was obtained from Invitrogen (France). For each assay, 100μl of infected cell culture supernatant was mixed with 100μl of stabilizer solution to obtain final concentrations at 5% and 10% with sorbitol and trehalose, at 3.5% and 7% with sucrose, and at 25% and 50% with FBS.

### 3. Preparation of virus samples

Confluent Vero, MRC-5 and Hep-2 cells in 175-cm^2^ flasks were respectively infected with Chikungunya virus (CHIKV), Herpesvirus type 1 (HSV-1), Human Echovirus 13 (EV-13) or Human Adenovirus type C (HAdV-C) with a multiplicity of infection (MOI) between 0.1 and 0.01. When a 50% cytopathic effect (CPE) was observed, cell supernatant media were clarified using low-speed centrifugation (1500g for 10 min) and supplemented with 25mM HEPES (Sigma). 200μL aliquots of the appropriate virus, in appropriate stabilizing media, were added to sterile 2-mL lyophilization vials (Dutsher, France) and the aliquots were stored at -70°C prior to freeze drying.

### 4. Freeze drying of virus preparation

Lyophilization was carried out in a BSL3 laboratory using a CHRIST alpha 1–4 LSC freeze-dryer. To avoid cross contamination and to ensure identical conditions, each freeze-dried batch production was performed on a single virus strain with different stabilizer formulations. Briefly, as described in section *3*, 200μl of viral preparation were dispensed in sterile 2mL vials partially sealed with vented rubber stoppers before freezing in a dry ice/ethanol cooling bath.

Two lyophilization programs were used for comparison; (i) LP1 "optimized;", this is used routinely in our laboratory and was previously developed based on a yellow fever 17 D vaccine virus model (data not shown), which consisted of a 6-hour primary drying step (1.03 mbar, -20°C for 160 min then -10°C for 135 min) followed by a secondary drying step (0.006 mbar, +20°C for 360 min), (ii) LP2 "rapid"; this second program consisted of a single 6-hour secondary drying step (0.006mbar, +20°C). All the vials were finally sealed under vacuum following the lyophilization stage.

### 5. Storage and thermostability of freeze dried viruses

Separate freeze dried virus vials were exposed to different storage temperatures (-20°C, +4°C and +20°C) in a light-protected environment for 7 days. Vials were then rehydrated with 200μL of sterile distilled water before virus titration as described below.

To summarize and to simplify the comparison of the different freeze dried batches, the following abbreviation was adopted: LP1/2 (-20/+4/+20°C) for all samples freeze dried using the LP1 or LP2 protocol and stored for one week at -20, +4 or +20°C. Storage at -20°C represents the laboratory standard for EVA collection preservation of freeze dried viruses. A storage at +4°C or +20°C for one week mimics conditions which can be encountered during sample transportation from the EVA to the customer laboratory.

### 6. Virus titration and titre comparison

Virus infectivity was quantified by estimating the 50% tissue culture infectivity dose (TCID_50_) using standard cell culture procedures. Briefly, when cells reached 80% confluence in 96-well microtitre plates, six replicates were infected with 150μL of tenfold serial dilutions of the virus sample, and then incubated for 7 days at 37°C in a humidified atmosphere of 5% CO2. CPE was monitored using an inverted microscope, and infectivity titres were expressed as TCID_50_/ml based on the Karber formula. The virus infectivity was then compared and analyzed to determine the optimal sample formulation under the different conditions of preparation and storage. Moreover, to evaluate the program and temperature influence on viral infectivity independently of sample formulation, *t*-tests were used to estimate the difference between groups of data which were expressed as mean virus titres ± standard deviations (Table 2).

**Table 2:** Mean and standard deviations of viral formulation titres obtained for each protocol and each storage temperature.

Virus	Process	Strorage temperature	Average of viral titers (Log_10_ TCID_50_/ml)	± Standard deviation
Chikungunya virus	LP1	+20°C	6.20	0.36
		+4°C	7.80	1.30
		-20°C	8.00	0.86
	LP2	+20°C	6.60	0.30
		+4°C	7.20	0.60
		-20°C	6.86	0.20
Herpesvirus type 1	LP1	+20°C	7.20	0.90
		+4°C	6.70	0.50
		-20°C	6.90	0.20
	LP2	+20°C	7.20	1.50
		+4°C	6.70	0.40
		-20°C	9.20	1.30
Human Echovirus 13	LP1	+20°C	2.00	1.70
		+4°C	3.30	1.00
		-20°C	3.60	0.70
	LP2	+20°C	2.00	1.50
		+4°C	3.10	1.00
		-20°C	3.40	0.90
Human Adenovirus type C	LP1	+20°C	3.00	1.00
		+4°C	3.80	0.34
		-20°C	4.10	0.30
	LP2	+20°C	2.60	1.10
		+4°C	3.60	0.20
		-20°C	3.40	0.90

Independent of the sample formulation, *t*-tests were used to estimate the difference between groups of data which were expressed as mean virus titres ± standard deviations.

## Results

Table 1 identifies the four representative viruses used in this study. Following the procedures described above, comparative infectivity levels were assessed for each of these viruses, under the different freeze drying protocols (LP1, LP2) identified in Table 2 and after storage for one week at the different temperatures.

### Chikungunya virus

**Fig 1:**
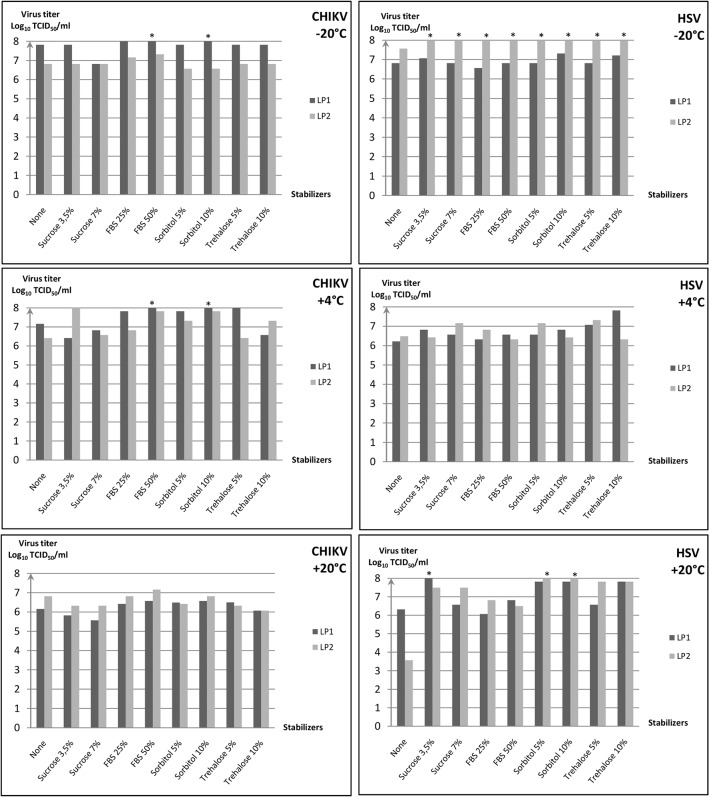
Enveloped virus infectivity recovery after lyophilization. Chikungunya virus and Herpesvirus type 1 were freeze dried with/without different stabilizers using two different protocols (LP1 and LP2). After seven days at different temperature storage (-20°C, +4°C, +20°C), infectivity of each virus formulation were determinate by TCID_50_ assays. Chikungunya virus infectivity after storage at -20°C, +4°C, +20°C. Herpesvirus type 1 infectivity after storage at -20°C, +4°C, +20°C.* TCID50/ml >10^8^.

Fig 1 describes TCID_50_ data obtained for CHIKV.

Independently of sample formulation, recovered virus infectivity levels were significantly higher (p<0.004) for LP1 (+4°C) (mean viral titre of 10^7.8±1.3^ TCID_50_/ml) and LP1 (-20°C) freeze dried samples (mean viral titre of 10^8±0.9^ TCID_50_/ml), when compared with LP1 (+20°C). Also, LP2 (+20°C) produced similar yields of virus. (p = 0.07).

Moreover, independently of sample formulation, there were no significant differences for recovered virus infectivity in the LP2 freeze dried samples in relation to the storage temperature and only slight differences in viral infectivity were observed between the various stabilizers by comparison with the mean viral titres for each storage temperature (10^6.6±0.3^ TCID_50_/ml at +20°C, 10^7.2±0.6^ TCID_50_/ml at +4°C and 10^6.9±0.2^ TCID_50_/ml at -20°C). However, for LP1 (-20°C) freeze dried samples, the used of FBS 50% significantly increased the mean viral titre by about 2 log_10_ TCID_50_ (10^8±0.9^ TCID_50_/ml versus 10^10.1^ TCID_50_/ml). For LP1 (+4°C) freeze dried samples, formulated with sorbitol 10% resulted in recovery of the highest virus infectivity (more than 2 log_10_ TCID_50_ higher when compared with the mean viral titre 10^7.8±1.3^ TCID_50_/ml in the absence of sorbitol).

To summarize, a comparison of the results obtained for chikungunya virus, under all of the conditions described, revealed that the highest viral titres were obtained for LP1 (+4°C/-20°C) freeze dried samples, with 10% sorbitol or 50% FBS as stabilizers whereas the lowest viral titres were obtained for both LP1 and LP2 (+20°C) freeze dried samples with no significant difference between the stabilizers. The LP2 (-20°C) is not significantly different from the LP2 (+20°C) result.

### Herpesvirus type 1

Fig 1 describes the TCID_50_ data obtained for HSV-1.

The results show that independently of sample formulation and storage temperature, there were no significant differences between the mean viral infectivity observed using the LP1 protocol (p>0.2). However, infectivity recovered was significantly higher (p<0.0001) when the LP2 freeze drying protocol was used and the samples were stored at -20°C (mean viral titre of 10^9.2±1.3^ TCID_50_/ml). Virus infectivity recovered was also statistically higher (p = 0.004) for the LP2 freeze-drying protocol when samples were stored at -20°C (mean viral titre of about 10^7.8±1.3^ TCID_50_/ml). These results were independent of the sample formulation and they refer to comparison with the mean viral titre obtained following storage at +20°C (10^7.2±1.5^ TCID_50_/ml) or +4°C (10^6.7±0.4^ TCID_50_/ml)_._ Moreover for the same samples, the most efficient stabilizers were 10% sorbitol and 5–10% trehalose with a gain in viral infectivity of about 1–2 log_10_ TCID_50_/ml by comparison with the mean viral titre (10^9.2±1.3^ TCID_50_/ml).

To summarize, the highest mean viral titres were obtained using the LP2 (-20°C) conditions (10^9.2±1.3^ TCID_50_/ml). Inversely, the lowest virus infectivity was obtained for LP2 (+20°C) freeze dried samples without stabilizer. However, under sub-optimal storage conditions (*i*.*e*. after one week at +20°C), 10% sorbitol combined with the LP2 protocol and 3.5% sucrose combined with the LP1 protocol were the more efficient stabilizers, increasing recovered viral titres by about 2 log_10_ TCID_50_ when compared with other freeze drying formulations.

### Human Echovirus 13

**Fig 2:**
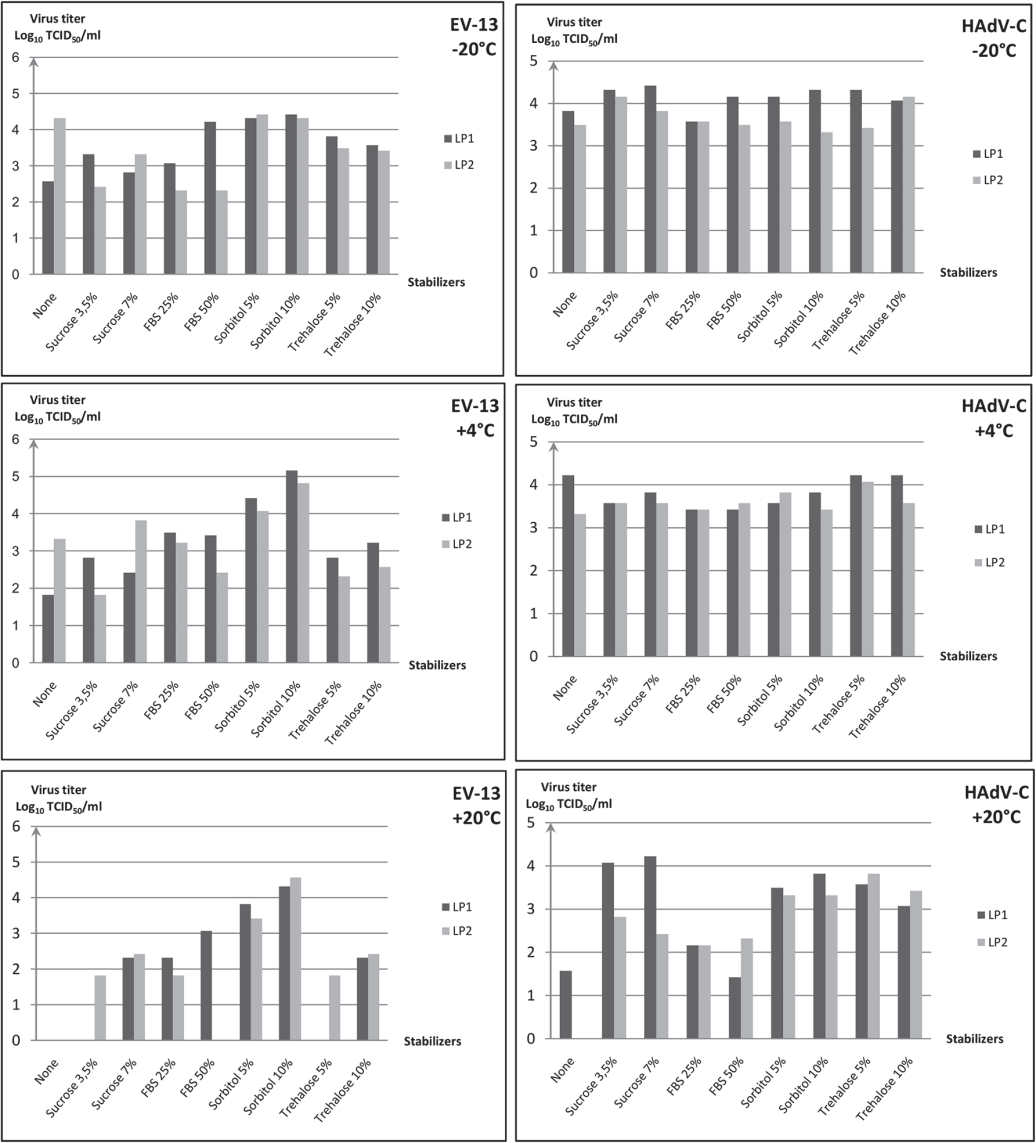
Non enveloped virus infectivity recovery after lyophilization. Human Echovirus 13 and Human Adenovirus type C were freeze dried with/without different stabilizers using two different protocols (LP1 and LP2). After seven days at different temperature storage (-20°C, +4°C, +20°C), infectivity of each virus formulation were determinate by TCID_50_ assays. Human Echovirus 13 infectivity after storage at -20°C, +4°C, +20°C. Human Adenovirus type C infectivity after storage at -20°C, +4°C, +20°C.

Fig 2 describes the TCID_50_ data obtained for EV-13.

Freeze dried human echovirus 13 appeared to be very unstable at +20°C, as virus viability was reduced to a very low level after one week at +20°C regardless of the freeze drying protocols and formulations. However, at +4°C and -20°C, 5–10% sorbitol proved to be an efficient stabilizer by preserving viral infectivity independent of the freeze-drying protocol.

The two lyophilization protocols (LP1 and LP2) produced closely similar results with no significant difference of mean virus titres recovered (p>0.05) at each storage temperature (ie +20°C, +4°C, -20°C). Independently the sample formulation and by comparison with the mean viral titres obtained at +20°C (10^2±1.5^ TCID_50_/ml), the recovery of virus infectivity was significantly higher (p = 0.03) for LP2 (-20°C) freeze dried samples (mean viral titre of about 10^3.4±0.9^ TCID_50_/ml)_._ Similarly and by comparison with the mean viral titres obtained at +20°C (10^2±1.7^ TCID_50_/ml), the recovery of virus infectivity was significantly higher (p = 0.02) for LP1 (-20°C) freeze dried virus (mean viral titre 10^3.6±0.7^ TCID_50_/ml).

To summarize, the highest mean viral titres were obtained using either the LP1 or LP2 protocol following storage at low temperatures +4°C/-20°C. Moreover, the highest infectivity recovered was obtained for LP1 or LP2 (+4°C) freeze dried samples with formulations containing 5–10% sorbitol (increase of about 2 log_10_ TCID_50_ by comparison with other freeze dried formulations). Accordingly, the lowest mean virus infectivities were obtained for both LP1 and LP2 (+20°C) freeze-dried samples. Under sub-optimal storage conditions (*i*.*e*. after one week at +20°C), 5–10% sorbitol with the LP1 or LP2 protocol was also the more efficient stabilizer by increasing the viral titre recovery of about 2 log_10_ TCID_50_ by comparison with other freeze drying formulations.

### Human Adenovirus type C

Fig 2 describes the TCID_50_ data obtained for HAdV-C.

Independently of freeze drying formulations and lyophilization protocols, no significant differences were observed by comparing the mean viral infectivity for samples stored at +4°C or +20°C (p>0.05 in all cases). On the other hand, the virus recovery infectivity was statistically higher (p<0.05) for LP1 (-20°C) freeze dried samples (mean viral titre of 10^4.1±0.3^ TCID_50_/ml)_._

Under optimal storage conditions at +4°C/-20°C, no significant differences in viral infectivity recovery were observed from all the samples formulation (p>0.05). However, under sub-optimal conditions (one week at +20°C), sucrose 3.5 or 7% in combination with the LP1 lyophilization protocol was the more efficient stabilizer by comparison with other freeze dried formulations. This formulation produced a viral titre recovery as high as those obtained under optimal storage conditions.

## Discussion and Conclusion

A fundamental problem in preservation of freezed-dried live viruses is the possible loss of infectivity (i) either during the process of lyophilization (ii) or because of improper observation of the cold chain integrity often encountered during the transportation of viruses between laboratories [10]. There are unlimited parameters to consider during the freeze drying protocol that can affect the quality of the end product. Included among the many variables affecting the recovery of live virus from freeze-dried preparation: virus type and concentration; use of cryoprotectant; rates of freezing; volume, size and type of storage container; temperature; pH; and osmolality [11]. These factors interact interdependently to influence viral infectivity, which is best preserved when they are carefully controlled. The optimal changes are not only confined to the different material suspensions which all have varying collapse or eutectic points to be observed, but also to the lyophilization protocol. In fact, different freezing or drying rates can produce vastly different freeze-dried products, both in appearance and viability of the virus [9].

In this study, we have considered that -20°C was the optimal temperature for long-term storage. Experiments conducted after one-week storage at either +4°C or +20°C were assayed to simulate failure due to the breakdown in cold-chain maintenance possibly encountered during the shipping of vials containing freeze-dried viruses, and to test whether the type of freeze-drying protocol, and the nature of stabilizers might improve the stability of end products and their ability to resist to uncontrolled conditions.

Two different lyophilization protocols were tested in this study: (i) LP1 (12 hours with primary and secondary drying steps) which was the "reference" protocol usually described [2, 5, 9]; (ii) the rapid LP2 protocol (6 hours with only the secondary drying step) was well-adapted to high-throughput workflow required in the framework of a virus collection. For most of the virus and conditions, no obvious difference could be observed between LP1 and LP2 in terms of preservation of virus viability using our experimental conditions. The major differences were observed in the absence of stabilizer for ADV (+20°C) and HSV (+20°C) where the infectiveness with LP2 was lost or clearly reduced, respectively. In contrast, in the absence of stabilizer, LP2 appeared more prone to maintain infectiveness of the freeze-dried Echovirus 13 at -20°C and +4°C. Clearly, both LP1 and LP2 protocols beneficiated from the addition of stabilizers, most particularly in the +20°C storage conditions, obviously the most deleterious for virus titres and infectiveness. A global analysis shows that there are limited differences of the performances of LP1 and LP2 when storage is done at -20°C; moreover supplementation with the most efficient stabilizers help to further limit the differences observed between LP1 and LP2. This suggests that, in the context of an active collection of viruses that requires to prepare freeze-dried material daily, it would be recommended to use LP1 for proper long-term storage (-20°C), and that LP2 may be more adapted to vials prepared in the intention of shipping.

It is known that the level of virus viability after freeze drying varies according to the virus family, and the efficacy of the protective agent (stabilizer). For 20 years sugars such as trehalose and sucrose were reported to enhance the tolerance to desiccation through stabilization of membranes and proteins [9]. Sucrose and trehalose have improved the stability of PPR virus, camelpox virus or live-attenuated mumps vaccines during freeze drying [12–17]. Similarly, sucrose [0.2M] was already identified as a powerful stabilizer for lyophilized viruses leading to a better stability during long-term storage and transportation [6,11, 18]. Gelatin [0.5%] combined with 10%-lactose or 10%-trehalose was also commonly used for lyophilization of poultry vaccines in Korea [19]. In our study, the supplementation of freeze-dried formulations with sucrose or trehalose demonstrated higher virus titres for herpes simplex virus and adenovirus. FBS is commonly used for cryopreservation of cells, but is rarely reported for viruses. Even though 50%-FBS provided satisfactory results in our study, we do not recommend to use it because of potential contamination with microorganisms (due to its biological origin) and because its composition may vary greatly depending on the supplier [20].

Sorbitol is recommended as cryoprotectant during freezing and lyophilization of several viruses such as cytomegalovirus, varicella-zoster virus, herpes simplex virus, respiratory syncytial virus, and 17D Yellow fever vaccine) [10]. Others studies have demonstrated that only stabilizer formulations containing sorbitol, were able to stabilize the lyophilized live attenuated duck viral hepatitis virus vaccine or increase the thermal stability of some viruses, such as measles virus [19, 21]. Similarly, sorbitol-gelatin was described as an effective stabilizer in the preparation of freeze-dried suspensions of measles virus [21] and rinderpest virus [22]. Our results confirm that sorbitol was the most efficient stabilizers for the 4 model viruses, particularly in sub-optimal storage temperatures. Although there is no clear advantage conferred by any of the stabilizer when the sample is maintained at -20°C, it is obvious that most of them play a major role in virus viability preservation when storage temperature increases. Regarding this point, we would recommend to use the sorbitol in our experimental conditions because (i) it provides excellent results using indistinctly LP1 or LP2 protocols, (ii) it allows stabilization of our four model viruses.

Usually, enveloped viruses demonstrate greater lability during storage than those lacking envelopes. In fact, decreases in viral titre correlate with demonstrable changes since the viral envelope is assumed to be associated with viral infectivity [11]. Lyophilization of a heat-labile lipid-enveloped virus such as Herpes simplex virus pointed out that high concentration (27% w/v) of stabilizers could achieve up to 80% recovery of the viral titre [23]. In our study, similar results were observed with HSV. The 2 non enveloped virus models (enterovirus and adenovirus) were also very heat sensitive, which manifested through deleterious effects when stored at +20°C for one week. Interestingly, this phenomenon was not observed with CHIKV.

This study focused on optimizing both the protocol and formulation for the lyophilization of selected model viruses under routine collection production and distribution conditions. Reference (LP1) and “rapid” (LP2) lyophilization protocols were conducted with a variety of sugar-based excipients and under different temperatures storage for one week. Although there is the need for more detailed analysis specifically dedicated for each virus species, the aim of this study was to evaluate a rapid protocol applicable to a broad range of viruses. Regarding this objective we recommend to use the combination of LP2 protocol together with 10%-sorbitol supplementation when aiming at high throughput workflow for preparing material in the intention to distribution. For long-term storage, the LP1 reference protocol can be associated with 10%-sorbitol supplementation.

Finally, due to few data available, the results obtained using four relevant and model viruses (enveloped/non enveloped RNA/DNA viruses) would improve the freeze drying conditions needed for the development and the distribution of a large virus collection. Future developments would concern optimization of these methods for different virus families and evaluation of these protocols for perennial virus conservation at the -20°C reference temperature storage.
